# TIF1-gamma IgG2 isotype is not associated with malignancy in juvenile dermatomyositis patients

**DOI:** 10.1093/rheumatology/keae182

**Published:** 2024-03-18

**Authors:** Huong D Nguyen, Fabienne Jouen, Benoit Déchelotte, Nadège Cordel, Cyril Gitiaux, Christine Bodemer, Pierre Quartier, Alexandre Belot, Kathryn O’Brien, Dario Cancemi, Isabelle Melki, Nicole Fabien, Sarah Tansley, Olivier Boyer, Lucy R Wedderburn, Brigitte Bader-Meunier, UK JDM Cohort and Biomarker Study, Kate Armon, Louise Coke, Julie Cook, Amy Nichols, Liza McCann, Ian Roberts, Eileen Baildam, Louise Hanna, Olivia Lloyd, Susan Wadeson, Michelle Andrews, Phil Riley, Ann McGovern, Verna Cuthbert, Clive Ryder, Janis Scott, Beverley Thomas, Taunton Southwood, Eslam Al-Abadi, Ruth Howman, Sue Wyatt, Gillian Jackson, Mark Wood, Tania Amin, Vanessa VanRooyen, Deborah Burton, Louise Turner, Heather Rostron, Sarah Hanson, Joyce Davidson, Janet Gardner-Medwin, Neil Martin, Sue Ferguson, Liz Waxman, Michael Browne, Roisin Boyle, Emily Blyth, Mark Friswell, Helen Foster, Alison Swift, Sharmila Jandial, Vicky Stevenson, Debbie Wade, Ethan Sen, Eve Smith, Lisa Qiao, Stuart Watson, Claire Duong, Stephen Crulley, Andrew Davies, Miss Caroline Miller, Lynne Bell, Flora McErlane, Sunil Sampath, Josh Bennet, Sharon King, Helen Venning, Rangaraj Satyapal, Elizabeth Stretton, Mary Jordan, Ellen Mosley, Anna Frost, Lindsay Crate, Kishore Warrier, Stefanie Stafford, Brogan Wrest, Lucy Wedderburn, Clarissa Pilkington, Nathan Hasson, Muthana Al-Obadi, Giulia Varnier, Sandrine Lacassagne, Sue Maillard, Lauren Stone, Elizabeth Halkon, Virginia Brown, Audrey Juggins, Sally Smith, Sian Lunt, Elli Enayat, Hemlata Varsani, Laura Kassoumeri, Laura Beard, Katie Arnold, Yvonne Glackin, Stephanie Simou, Beverley Almeida, Kiran Nistala, Raquel Marques, Claire Deakin, Parichat Khaosut, Stefanie Dowle, Charalampia Papadopoulou, Shireena Yasin, Christina Boros, Meredyth Wilkinson, Chris Piper, Cerise Johnson-Moore, Lucy Marshall, Kathryn O’Brien, Emily Robinson, Dominic Igbelina, Polly Livermore, Socrates Varakliotis, Rosie Hamilton, Huong D Nguyen, Dario Cancemi, Kevin Murray, Coziana Ciurtin, John Ioannou, Caitlin Clifford, Linda Suffield, Laura Hennelly, Helen Lee, Sam Leach, Helen Smith, Anne-Marie McMahon, Heather Chisem, Jeanette Hall, Amy Huffenberger, Nick Wilkinson, Emma Inness, Eunice Kendall, David Mayers, Ruth Etherton, Danielle Miller, Kathryn Bailey, Jacqui Clinch, Natalie Fineman, Helen Pluess-Hall, Suzanne Sketchley, Melanie Marsh, Anna Fry, Maisy Dawkins-Lloyd, Mashal Asif, Joyce Davidson, Margaret Connon, Lindsay Vallance, Kirsty Haslam, Charlene Bass-Woodcock, Trudy Booth, Louise Akeroyd, Alice Leahy, Amy Collier, Rebecca Cutts, Emma Macleod, Hans De Graaf, Brian Davidson, Sarah Hartfree, Elizabeth Fofana, Lorena Caruana

**Affiliations:** Department of Infection, Immunity & Inflammation Research and Teaching Department, UCL Great Ormond Street Institute of Child Health, London, UK; Department of Immunology and Biotherapy, Univ. Rouen Normandie, INSERM, U1234, Rouen, France; Department of Immunology and Biotherapy, CHU de Rouen, Rouen, France; Department of Immunology and Biotherapy, Univ. Rouen Normandie, INSERM, U1234, Rouen, France; Department of Immunology and Biotherapy, CHU de Rouen, Rouen, France; Department of Immunology and Biotherapy, Univ. Rouen Normandie, INSERM, U1234, Rouen, France; Department of Dermatology and Clinical Immunology, Guadeloupe University Hospital, Pointe-à-Pitre, Guadeloupe; Department of Paediatric Immunology, Hematology and Rheumatology, Université Paris Cité, Paris, France; Department of Paediatric Neurophysiology, Necker-Enfants Malades Hospitals, Paris, France; Department of Paediatric Dermatology and Dermatology, Necker-Enfants Malades Hospitals, Paris, France; Department of Paediatric Immunology, Hematology and Rheumatology, Université Paris Cité, Paris, France; Department of Paediatric Hematology-Immunology and Rheumatology, Necker-Enfants Malades Hospitals, Paris, France; Department of Paediatric Rheumatology, Femme-Mère-Enfants Hospital, Lyon, France; Department of Infection, Immunity & Inflammation Research and Teaching Department, UCL Great Ormond Street Institute of Child Health, London, UK; Department of Infection, Immunity & Inflammation Research and Teaching Department, UCL Great Ormond Street Institute of Child Health, London, UK; Department of Paediatrics, Hôpital Robert Debré, Paris, France; Department of Immunology, Laboratory of Immunology, Hôpital Lyon Sud, Lyon, France; Royal National Hospital for Rheumatic Diseases, Royal United Hospital Bath NHS Trust, Bath, UK; Department of Life Sciences, University of Bath, Bath, UK; Department of Immunology and Biotherapy, Univ. Rouen Normandie, INSERM, U1234, Rouen, France; Department of Immunology and Biotherapy, CHU de Rouen, Rouen, France; Department of Infection, Immunity & Inflammation Research and Teaching Department, UCL Great Ormond Street Institute of Child Health, London, UK; NIHR Biomedical Research Centre at Great Ormond Street Hospital for Children, London, UK; Centre for Adolescent Rheumatology Versus Arthritis at UCL, UCLH and GOSH, London, UK; Department of Paediatric Immunology, Hematology and Rheumatology, Université Paris Cité, Paris, France; Department of Paediatric Hematology-Immunology and Rheumatology, Necker-Enfants Malades Hospitals, Paris, France

Rheumatology key message

- TIF1γ IgG2 is a biomarker for malignancy in adult DM but not in JDM.

Dear Editor, The disease presentation and associated complications of juvenile- and adult-onset dermatomyositis (JDM and adult DM) differ significantly. The pathological hallmarks of DM are similar between JDM and adult DM, including skin rashes and proximal muscle weakness; however, the prevalence and implication of associated autoantibodies varies depending on age of onset.

Myositis-specific antibodies (MSA) have been used as a prognostic tool to aid management of disease in both adult DM and JDM [1]. In JDM, a prevalent MSA is anti-transcription intermediary factor 1(anti-TIF1γ), which is the most common MSA in Caucasian patients. Although the clinical and pathological features of the anti-TIF1γ subtype are significantly heterogeneous [2, 3], this MSA has been well known to be associated with malignancy in adult DM [4]. More specifically, we have previously shown that adult DM patients with cancer have a significantly higher frequency and serological level of anti-TIF1γ-IgG2 isotype [4], which raises the question of whether there is also an association between anti-TIF1γ-IgG2 isotype and cancer in JDM. To explore this, we investigated all anti-TIF1γ isotypes and their associations with clinical manifestations in JDM.

We conducted a retrospective study of 31 patients to evaluate clinical features of anti-TIF1γ-positive patients from diagnosis to the most recent clinical visits (Supplementary Table S1, available at *Rheumatology* online). The median duration of follow-up was 6.6 years (min 1.0–max 20.6 years).

This cohort included 20 patients from French healthcare centres and 11 patients from the UK healthcare centres. Serum was collected near time of diagnosis or flare. Serum samples were first tested for anti-TIF1γ using either the commercial Myositis Profile 4 EUROLINE immunoblot (EUROIMMUN AG, Lübeck, Germany) or immunoprecipitation. Within those with anti-TIF1γ auto-antibodies, anti-TIF1γ isotypes including IgG1, IgG2, IgG3 and IgG4 were measured using a multiplex ALBIA assay developed by Aussy *et al.* [5]. The median duration from diagnosis date to sample date was 10.3 months (interquartile range 1.2–9.6).

Out of 31 children, 54.8% (17) were Caucasian, followed by North-African (Maghreb) (25.8%, *n* = 7) and other minority groups. Male to female ratio was 14/17. Average age at diagnosis was 6.8 ± 3.3 years. All 31 patients had IgG1 isotype, and 14/31 had more than one isotype of anti-TIF1γ. There was no mutual exclusion between the four isotypes, as various combinations of isotypes were found (Fig. 1A).

**Figure 1.**
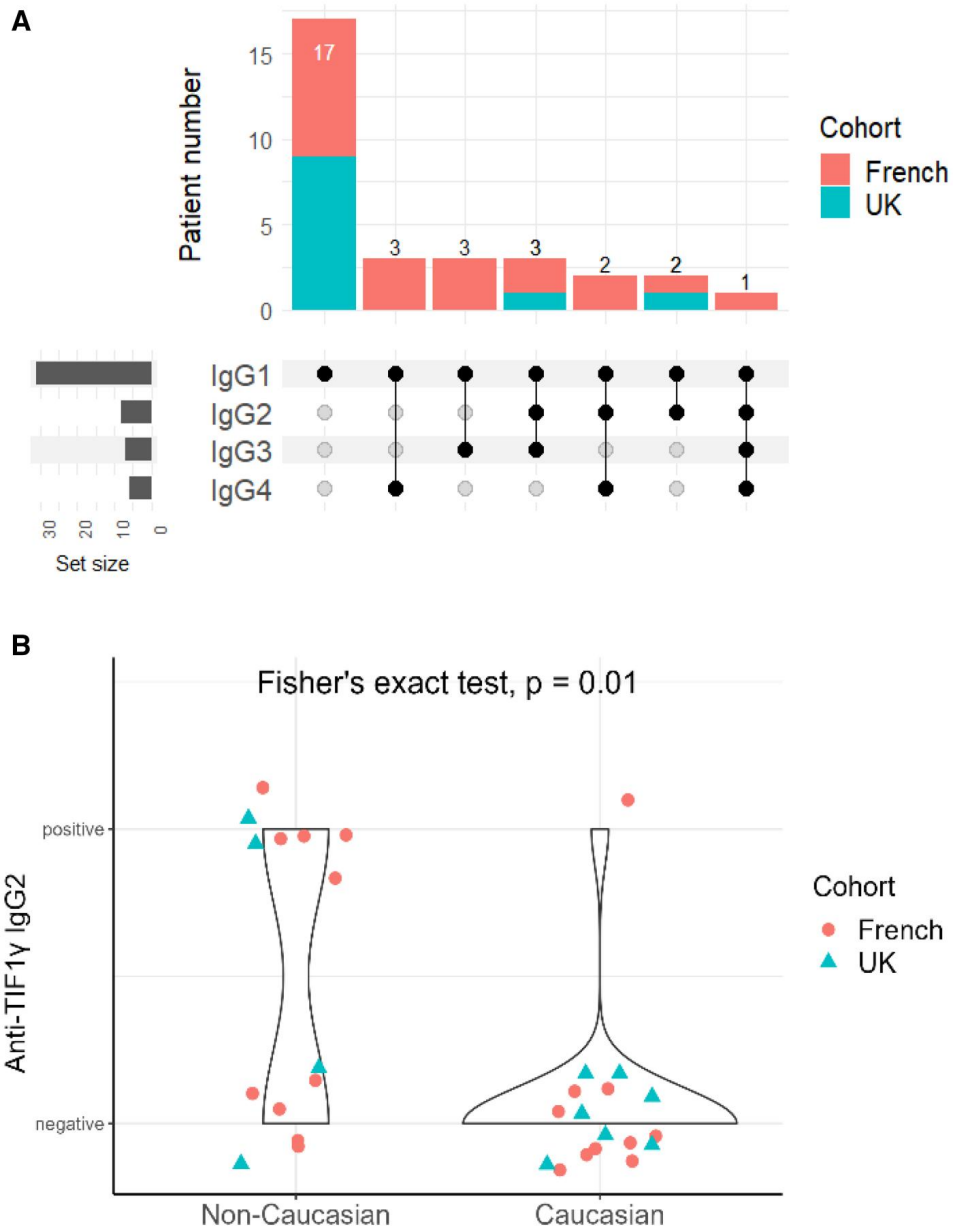
Detection of anti-TIF1γ auto-antibodies and demographic association in patients with JDM. Anti-TIF1γ auto-antibodies were measured by lineblot or immunoprecipitation. Anti-TIF1γ isotypes including IgG1, IgG2, IgG3 and IgG4 were measured using the multiplex ALBIA assay in both cohorts. (**A**) Diverse combination of anti-TIF1γ isotypes detected in JDM patients from French and UK cohorts. (**B**) Anti-TIF1γ IgG2 positive patients were analysed according to ethnicity: anti-TIF1γ IgG2 is more prevalent in non-Caucasian patients

Although the IgG2 isotype of anti-TIF1γ has been shown to be a biomarker for malignancy and mortality in adult DM, there was no report of malignancy in this paediatric cohort (Supplementary Table S1, available at *Rheumatology* online ). In our JDM cohort, the rate of IgG2-positive was 25.8% (8/31). We did not observe any difference in clinical presentation or outcome between IgG2-positive *vs* IgG2-negative patients.

Interestingly, there was a significant difference regarding anti-TIF1γ-IgG2 prevalence between ethnic groups (Kruskal–Wallis’s test, *P* = 0.01, Supplementary Fig. S1, available at *Rheumatology* online). Specifically, although Caucasian patients were the majority (17/31, 54.8%) of this cohort, only 1/8 (12.5%) IgG2-positive cases was Caucasian, which made the IgG2 prevalence significantly different from non-Caucasian population (Fisher’s exact test, *P* = 0.01) (Fig. 1B). Notably, 4/8 IgG2-positive patients (50%) were found in North-African (Maghreb) population, making up 44.4% (4/9) of this ethnic group (Supplementary Fig. S1, available at *Rheumatology* online).

We also observed that anti-TIF1γ isotypes can change over time. Specifically, of six patients tested for anti-TIF1γ isotypes at a second time point, four cases had changes in serological levels of anti-TIF1γ isotypes: two had lower titre levels, one lost positive status for IgG2 and IgG3, and one gained positive status for IgG4. Average time duration between the first and second sample time-points was 18.7 ± 13.4 months. Further investigation in larger cohorts is needed to clarify whether the changes in isotype titre are age-dependent or correlated to treatment response.

Two French patients in this JDM cohort died from persistently severe JDM which led to multi-organ failure despite being treated with CS, MTX, MMF, rituximab and plasma exchange. Both patients were positive for IgG4 but negative for IgG2. Based on the analysis in the French cohort (as IgG4 was not detected in UK cohort), IgG4-positive patients might be more likely to have severe onset (Fisher’s exact test, *P* = 0.03) (Supplementary Fig. S2, available at *Rheumatology* online). Severity was defined according to previous consensus [6] by: (i) admission to intensive care unit, and/or the presence of (ii) skin ulcerations and/or (iii) severe muscle involvement, defined by Childhood Myositis Assessment Scale ≤15 or Manual Muscle Testing ≤30, and/or (iv) a severe organ involvement (e.g. cardiovascular, pulmonary or gastrointestinal involvement, dysphonia or dysphagia) within the first month of diagnosis. Larger sample sizes are required to confirm a potential association of severe JDM onset with anti-TIF1γ-IgG4 isotype and the potential impact on disease management, if this association is confirmed.

In conclusion, our study shows the distribution and fluctuation of anti-TIF1γ isotypes in JDM patients. Our data indicated that there may be a relationship between anti-TIF1γ IgG2 isotype and ethnicity. Importantly, although IgG2 is a biomarker for cancer in adult DM, it is not associated with severe onset or manifestations such as mortality or malignancy in JDM patients, which is consistent with previous reports [7, 8].

## Supplementary Material

keae182_Supplementary_Data

## Data Availability

Data on the cohorts’ studies can be applied to through the corresponding author (L.R.W.).
