# Supplementary material for: TIF1-gamma IgG2 isotype is not associated with malignancy in juvenile dermatomyositis patients
Source: Rheumatology (Oxford). 2024 Mar 18;63(10):e281–4. doi: 10.1093/rheumatology/keae182 (PMC11443014; doi:10.1093/rheumatology/keae182)
Supplement: keae182_Supplementary_Data [file keae182_supplementary_data.docx]

**Nguyen et al, Supplemental Materials**

**Supplementary Table S1**: Summary of clinical features of anti-TIF1γ-positive JDM patients.

|  | **Clinical features (n = 31)** | **Frequency** |
| --- | --- | --- |
| At time of serum sample | Skin rash | 96.8% (30) |
|  | Skin Gottron | 87.1% (27) |
|  | Skin ulceration | 12.9% (4) |
|  | Skin oedema | 12.9% (4) |
|  | Calcinosis | 16.1% (5) |
|  | Nailfold change | 71.0% (22) |
|  | Malignancy | 0% (0) |
|  | Joint involvement | 29.9% (9) |
|  | Lung involvement | 6.5%(2) |
|  | Gastrointestinal involvement | 6.5%(2) |
|  | Dysphonia | 12.9% (4) |
|  | Dysphagia | 16.1% (5) |
| From diagnosis to the last follow-up visit. | Responded to methotrexate | 51.6% (16) |
|  | Severe manifestation | 22.6% (7) |
|  | Skin remission (skin-DAS ≤1, no ulcerations or erythema) | 48.4% (15) |
|  | Muscle remission (CMAS ≥48 & MMT8 ≥78) | 67.7% (21) |
|  | Deceased | 6.5% (2) |

**
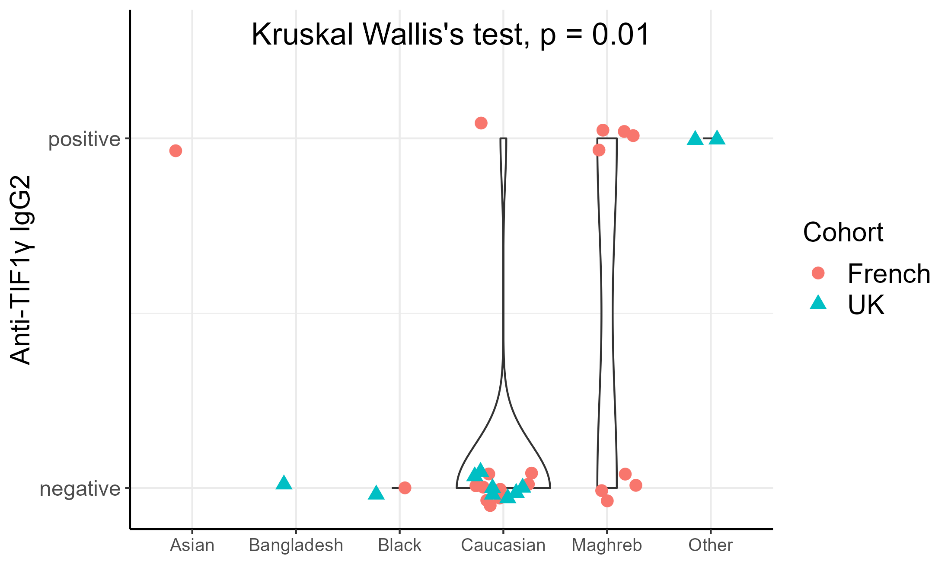
**

**Supplementary Figure S1**. Prevalence of anti-TIF1γ IgG2 isotype in different ethnic groups.


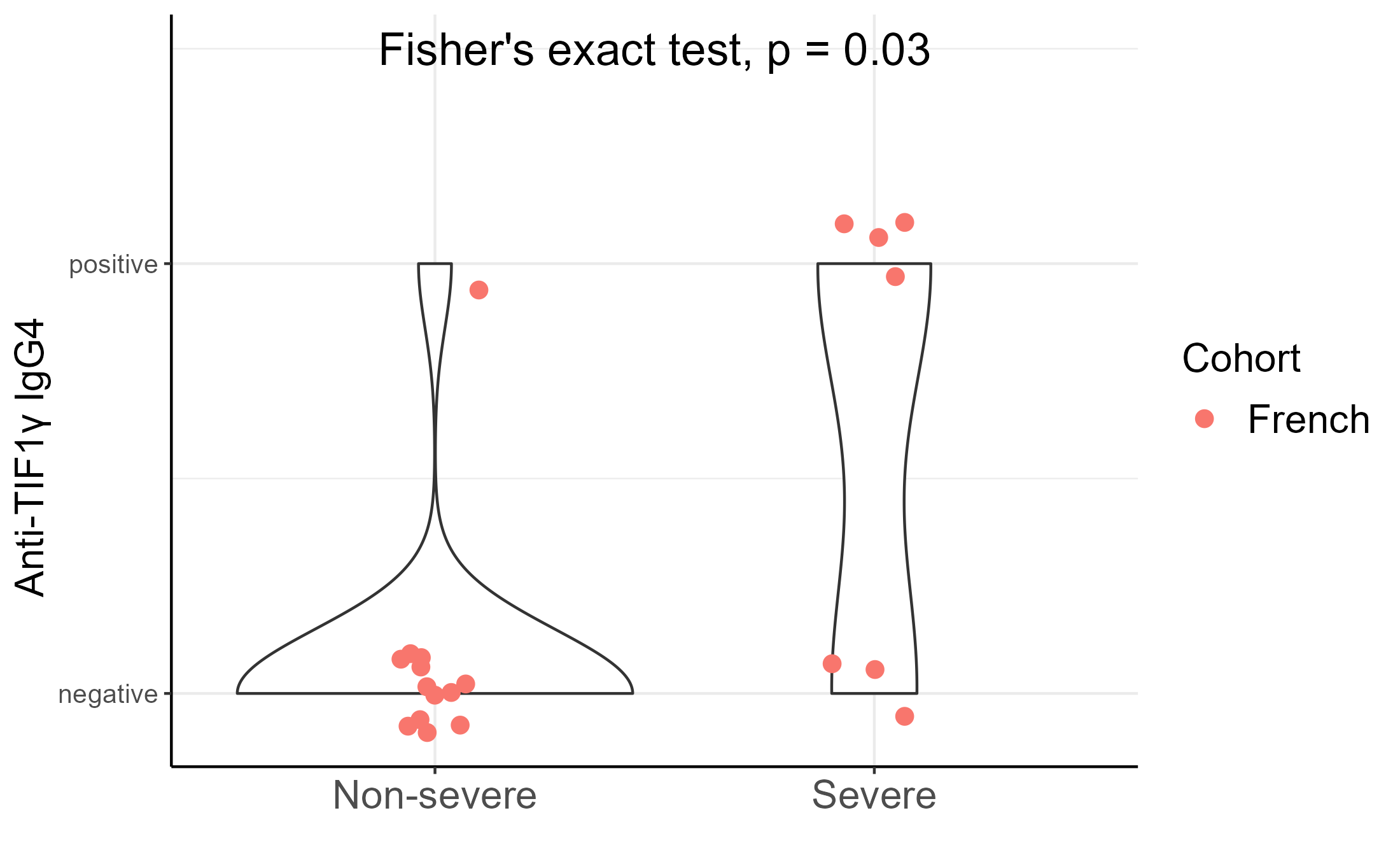


**Supplementary Figure S2**. IgG4 is more prevalent in French JDM patients with severe onset.
